# Dynamic Barrier Modulation in Graphene–Diamond Schottky Interfaces for Enhanced Ultraviolet Photodetection

**DOI:** 10.3390/s25247536

**Published:** 2025-12-11

**Authors:** Xiaohui Zhang, Kang Liu, Saifei Fan, Sen Zhang, Fei Xia, Benjian Liu, Bing Dai, Yumin Zhang, Jiaqi Zhu

**Affiliations:** 1 National Key Laboratory of Science and Technology on Advanced Composites in Special Environments, Harbin Institute of Technology, Harbin 150080, China; 2 Zhengzhou Research Institute of Harbin Institute of Technology, Zhengzhou 450046, China; 3 Southwest Institute of Technical Physics, Chengdu 610041, China; 4 Key Laboratory of Micro-Systems and Micro-Structures Manufacturing, Ministry of Education, Harbin 150080, China; 5 HRG Institute (Zhongshan) of Unmanned Equipment & AI, Zhongshan 528400, China

**Keywords:** diamond, graphene, UV photodetector, barrier photo-modulation, optical communication

## Abstract

Wide-bandgap diamond photodetectors face a fundamental trade-off between dark current suppression and photocurrent collection due to high Schottky barriers. Here, a photo-modulation strategy is demonstrated by integrating monolayer graphene as transparent electrodes on oxygen-terminated single-crystal diamond. The atomically thin graphene (87.3% UV transmittance at 220 nm) allows photons to penetrate and dynamically reduce Schottky barriers through photoinduced electric fields, while maintaining high barriers (~2.3 eV) under dark conditions for ultralow leakage current. Compared with conventional 100 nm Au electrodes, graphene-based devices exhibit a 4.9-fold responsivity improvement (0.158 A/W at 220 nm) and a 5.2-fold detectivity increase (8.35 × 10^13^ cm·Hz^1/2^/W), while preserving ultralow dark current (~10^−12^ A at ±100 V). XPS measurements confirm a minimal Fermi level shift (0.06 eV) upon graphene integration, demonstrating robust surface state pinning by oxygen termination. Transient photoresponse reveals a 27% faster rise time (30 ns vs. 41 ns) with bi-exponential decay governed by band-to-band recombination (τ1 ≈ 75 ns) and trap-assisted recombination (τ2 ≈ 411 ns). The devices maintain stable performance after one month of ambient exposure and successfully demonstrate UV optical communication capability. This transparent electrode approach offers a versatile strategy for enhancing wide-bandgap semiconductor photodetectors for secure communications, environmental monitoring, and industrial sensing applications.

## 1. Introduction

Single-crystal diamond has emerged as a premier material for deep ultraviolet (UV) photodetectors operating in the solar-blind region, where atmospheric absorption of solar radiation enables high signal-to-noise ratio detection [1]. Diamond’s exceptional properties—ultrawide bandgap (5.5 eV), high carrier mobility (~4500 cm^2^/V·s for electrons), superior thermal conductivity (~2200 W/m·K), and remarkable radiation hardness [2,3]—make it uniquely suited for demanding applications including secure communications [4], flame detection [5,6], environmental monitoring [7,8], and space-based astronomy [9,10]. Unlike conventional UV photodetectors based on AlGaN or SiC, diamond devices can operate reliably in extreme environments with high temperatures, intense radiation fields, and corrosive atmospheres while maintaining excellent photoresponse characteristics [11,12,13].

Despite these advantages, diamond UV photodetectors face a fundamental challenge in balancing dark current suppression with efficient photocurrent collection. The lack of reliable shallow n-type doping technology, due to the deep energy levels of nitrogen and phosphorus dopants in diamond’s wide bandgap, precludes the fabrication of p-n junction structures [14,15,16]. Consequently, photodetectors with metal–semiconductor–metal (MSM) configurations have become the dominant architecture [17,18,19,20]. While high Schottky barriers effectively suppress dark current to ultralow levels, they simultaneously hinder photogenerated carrier extraction, creating an inherent performance trade-off. This limitation is exacerbated by the pronounced Fermi level pinning at oxygen-terminated diamond surfaces, where high-density surface acceptor states result in nearly constant barrier heights regardless of metal work function [21], rendering conventional barrier engineering strategies ineffective [22,23,24]. It is well established that photovoltaic effects reduce junction barriers under illumination [25,26]. For narrow-bandgap materials such as silicon and germanium, the relatively small Schottky barriers result in modest photovoltaic modulation. This high barrier suggests that diamond can be as sensitive to light as p-n junctions in narrow-bandgap materials, exhibiting significant barrier reduction under UV illumination. Crucially, this large modulation window provides substantial operating space for barrier photo-regulation mechanisms. An alternative strategy therefore involves dynamic barrier modulation rather than static barrier engineering. Our previous work validated this light-modulated Schottky barrier mechanism in diamond photodetectors [27]. However, limited by the UV transmittance of metal electrodes, performance enhancement remains to be further explored.

Monolayer graphene has emerged as an exceptional transparent electrode material, combining extraordinarily high UV transparency with excellent electrical conductivity, mechanical flexibility, and chemical stability [28,29]. These properties make graphene particularly attractive for applications requiring both high optical transmittance and reliable electrical contact. Despite these compelling advantages, the graphene–diamond heterojunction remains largely unexplored for UV photodetection. Previous work primarily focused on in situ phase-converted graphitic electrodes for photoconductor-type devices [30,31], while the potential of transparent monolayer graphene for Schottky barrier photo-modulation and high-speed applications such as UV optical communication remains underexplored and warrants further investigation. In this work, monolayer graphene is integrated as transparent electrodes on oxygen-terminated single-crystal diamond, achieving substantial photodetection enhancement through Schottky barrier photo-modulation. Compared with conventional 100 nm Au electrodes, graphene-based devices demonstrate 4.9-fold responsivity improvement (0.158 A/W at 220 nm) and 5.2-fold detectivity enhancement (8.35 × 10^13^ cm·Hz^1/2^/W) while maintaining ultralow dark current (~10^−12^ A at ±100 V). X-ray photoelectron spectroscopy reveals minimal Fermi level perturbation upon graphene integration, confirming robust surface state pinning. Transient measurements show a 27% faster response with bi-exponential decay characteristics. The practical utility is validated through a functional UV optical communication system. This transparent electrode strategy offers a versatile approach for enhancing wide-bandgap semiconductor photodetectors without complex device modifications.

## 2. Materials and Methods

**Figure 1:**
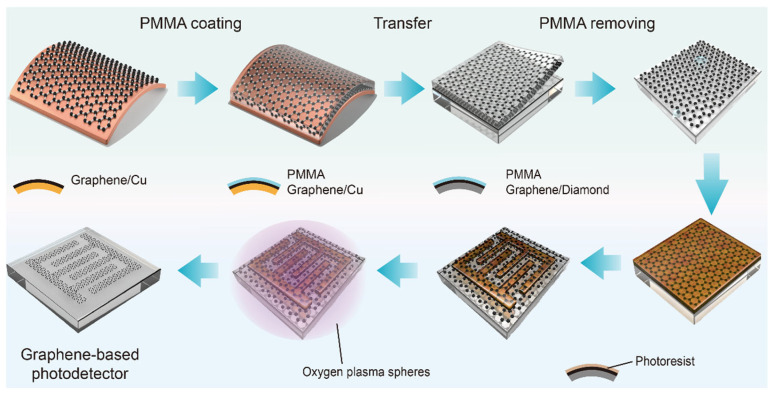
Fabrication process of graphene-based diamond UV photodetector. PMMA-assisted transfer of monolayer graphene onto oxygen-terminated diamond with pre-patterned Au electrodes, followed by oxygen plasma patterning to create interdigitated graphene electrodes.

**Figure 2:**
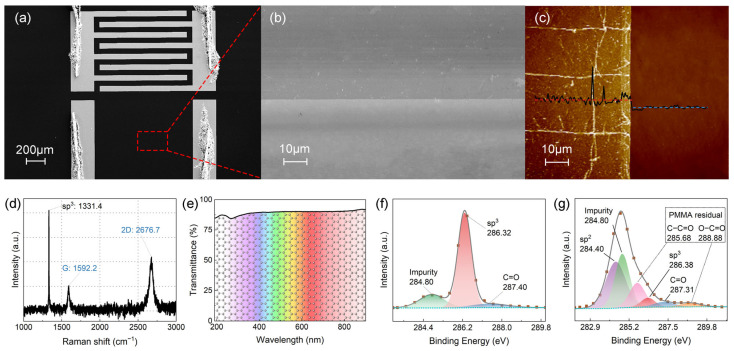
Morphological and optical characterization. (**a**,**b**) SEM images showing electrode architecture and graphene boundaries. (**c**) AFM topography confirming 1.6 nm thickness; red and blue dotted lines denote average thicknesses of graphene-covered and bare diamond regions, respectively. (**d**) Raman spectrum with I_2D_/I_G_ = 1.69. (**e**) UV-Vis transmittance exceeding 87% in the solar-blind region. (**f**,**g**) High-resolution XPS C 1s spectra of bare and graphene-covered oxygen-terminated diamond.

Single-crystal diamond substrates (Element Six, Santa Clara, CA, USA, (100) orientation, 3 × 3 × 0.5 mm^3^ [N] < 5 ppb) were ultrasonically cleaned in acetone, DI water, and isopropanol (15 min each) and then oxygen-terminated through mixed acid treatment (H_2_SO_4_:HNO_3_ = 3:1, 150 °C, 2 h). Interdigitated Au electrodes (100 nm thickness, 60 μm width/spacing, 1 mm length, effective area 0.47 mm^2^) were fabricated via photolithography and magnetron sputtering. Monolayer graphene on Cu foil (Hefei Vigon Material Technology, Hefei, China) was transferred using a PMMA-assisted wet transfer method: PMMA was spin-coated onto graphene/Cu, followed by Cu etching in 0.5 M FeCl_3_ solution and thorough DI water rinsing. The PMMA/graphene film was then transferred onto the pre-patterned diamond substrate, with PMMA subsequently removed in acetone. Vacuum annealing at 200 °C for 2 h eliminated residual organics. A second photolithography step with oxygen plasma etching (50 W, 20 sccm O_2_, 30 s) defined the graphene electrode patterns, creating the final device architecture with both graphene and Au electrode pairs for direct performance comparison. The resulting structure in the pad region, from bottom to top, consists of diamond, oxygen termination, Au, and graphene. Importantly, since PMMA is coated on the top surface of graphene during transfer, the graphene–diamond interface in the photoactive region remains free of PMMA contamination. The complete fabrication process is illustrated in Figure 1, and the resulting device structure is shown in Figure 2a.

Morphology was characterized using SEM (Sigma 300, Carl Zeiss AG, Oberkochen, Germany) and AFM (Dimension ICON, Bruker, Billerica, MA, USA; tapping mode). Raman spectroscopy was performed using a LabRAM HR Evolution (HORIBA, Kyoto, Japan) with 532 nm excitation. Optical transmittance was measured using a UV-Vis spectrophotometer (TU-1901, PERSEE, Beijing, China)). Electronic structure was analyzed via XPS (Thermo Fisher Scientific, Waltham, MA, USA; Al Kα source). Electrical measurements employed a Keithley 4200-SCS (TEKTRONIX, Beaverton, OR, USA) parameter analyzer. Photoresponse was evaluated using two light sources: a deuterium lamp (DH-2000, Ocean Insight, Orlando, FL, USA, 200–400 nm) for qualitative assessment and a 1000 W xenon lamp with monochromator (LE-LPM-HS211, LEOPTICS, Shenzhen, China) for spectral responsivity measurements (200–1100 nm). Optical power was calibrated using a power meter (LE-LPM-HS211AC, LEOPTICS, Shenzhen, China). Temporal response was measured using a 213 nm pulsed laser (LE-LS-213-20QFB, LEOPTICS, Shenzhen, China, 10 ns pulse width) with signals recorded on a digital oscilloscope (MDO3102, Tektronix, Beaverton, OR, USA). All measurements were performed at room temperature under ambient conditions.

## 3. Results and Discussion

Figure 2 presents the morphological and optical characterization of the fabricated devices. SEM imaging reveals the interdigitated electrode architecture with clear demarcation between graphene and exposed diamond regions (Figure 2a,b). The sharp boundaries produced by oxygen plasma etching demonstrate precise pattern transfer without edge damage. AFM measurements indicate a graphene thickness of ~1.6 nm (Figure 2c), exceeding the theoretical monolayer value (0.34 nm) due to residual PMMA and interfacial water layers, consistent with wet-transferred graphene on various substrates [32,33]. Since PMMA resides on the graphene top surface rather than at the graphene–diamond interface, it does not affect the Schottky junction properties but may introduce minor series resistance in the carrier transport path. Raman spectroscopy performed on Au pads—to avoid diamond signal interference—exhibits characteristic graphene peaks: G (1592.2 cm^−1^), 2D (2676.7 cm^−1^), with I_2D_/I_G_ ≈ 1.69 confirming the monolayer nature (Figure 2d). The peak at 1331.4 cm^−1^ originates from the underlying diamond substrate. UV-Vis transmittance measurements reveal an exceptional transparency of 87–95% across 200–900 nm (Figure 2e). The transmittance exhibits a gradual decrease toward shorter wavelengths due to enhanced interband π→π* transitions, maintaining 87.3% at 220 nm where diamond shows peak photoresponse. The preservation of such high transmittance at diamond’s peak photoresponse wavelength (220–225 nm) is critical for enabling efficient photo-modulation at the graphene–diamond interface, as it allows substantial UV photon flux to reach the Schottky junction region where carrier generation and barrier modulation occur. The graphene–diamond interface was investigated through XPS analysis. High-resolution C 1s spectra of bare oxygen-terminated diamond (Figure 2f) show a dominant sp3 peak at 286.32 eV with a C=O component at 287.40 eV, confirming effective oxygen termination. Upon graphene transfer (Figure 2g), a distinct sp^2^ peak emerges at 284.40 eV while the diamond sp^3^ peak shifts minimally to 286.38 eV (Δ = 0.06 eV). Additional peaks at 285.68 eV and 288.88 eV correspond to residual PMMA contamination.

**Figure 3:**
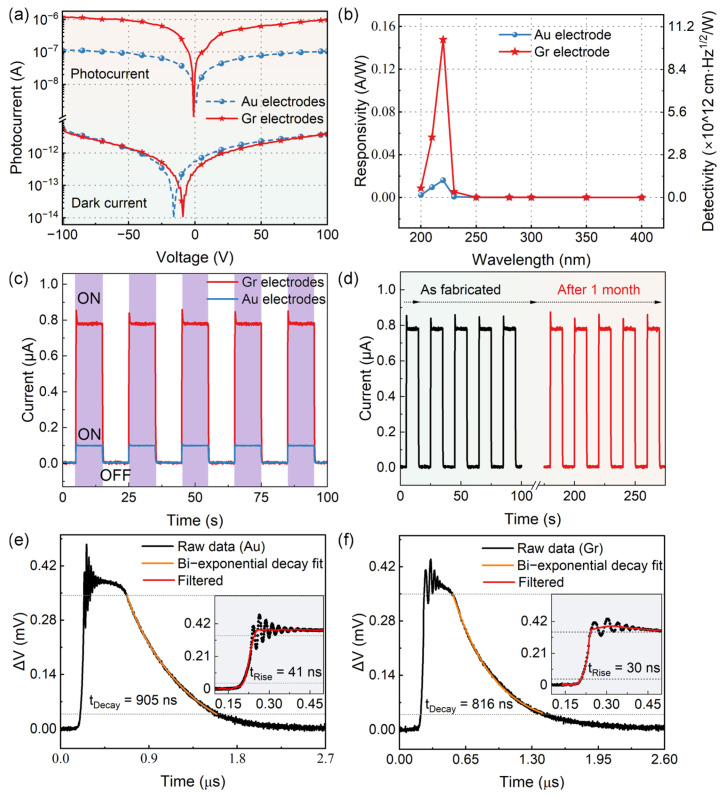
Performance comparison of graphene versus Au electrode photodetectors. (**a**) I–V characteristics showing enhanced photocurrent with preserved dark current. (**b**) Spectral responsivity and detectivity peaking at 220 nm. (**c**) Stable on/off switching under pulsed illumination. (**d**) Long-term stability over one month. (**e**,**f**) Transient photoresponse showing faster rise time for graphene (30 ns) versus Au (41 ns) electrodes.

Figure 3 compares the photodetection performance of graphene and Au electrode devices. Under dark conditions, both configurations exhibit nearly identical I–V characteristics with ultralow current (~10^−12^ A at ±100 V), confirming that the oxygen-terminated diamond surface state pinning dominates barrier formation regardless of electrode material (Figure 3a). However, under deuterium lamp illumination, the graphene-electrode device demonstrates a substantially enhanced photocurrent, reaching 8.85 × 10^−7^ A compared with 1.13 × 10^−7^ A for Au electrodes at 100 V—a 7.8-fold enhancement. Spectral responsivity measurements reveal peak performance at 220 nm, with graphene electrodes achieving 0.158 A/W versus 0.032 A/W for Au, representing a 4.9-fold improvement (Figure 3b). The responsivity

R = I/P,(1)

is calculated using the photocurrent I and incident optical power P (corrected for wavelength-dependent graphene transmittance T(λ)) [34]. The specific detectivity

(2) $$D^{*}=RA^{1/2}/{(2qI_{\mathrm{dark}})}^{1/2},$$

which characterizes the detector’s ability to detect weak optical signals in the presence of noise [34], correspondingly increases from 1.62 × 10^13^ to 8.35 × 10^13^ cm·Hz^1/2^/W. Here, A is the effective detection area; q is the elementary charge, and I_dark_ is the dark current.

Time-resolved measurements at 50 V bias under deuterium lamp illumination (Ocean Optics DH-2000, 25 W) demonstrate stable on/off switching behavior (Figure 3c) with excellent long-term stability—negligible degradation after one month of ambient exposure (Figure 3d), attributed to the chemical inertness of both graphene and diamond. Transient photoresponse analysis using 213 nm pulsed laser excitation reveals improved temporal characteristics: the graphene device exhibits $\tau_{\mathrm{rise}}$ = 30 ns compared with 41 ns for Au electrodes, a 27% improvement (Figure 3e,f). Both devices show bi-exponential decay behavior following the standard form [35,36,37]:

(3) $$∆V\left(t\right)=A_{1}\exp(-\frac{t}{\tau_{1}})+A_{2}\exp(-\frac{t}{\tau_{2}})+{∆V}_{0}$$

where A_1_ and A_2_ are amplitude coefficients; τ_1_ and τ_2_ are decay time constants, and ΔV_0_ is the baseline offset. Fitting of the experimental data yielded τ1 ≈ 75 ns and τ2 ≈ 411 ns for both electrode types, with goodness-of-fit R2 > 0.99. The bi-exponential model was selected based on its physical relevance to diamond photodetectors and superior fitting quality compared with single-exponential functions. The fast component corresponds to band-to-band recombination in diamond, while the slow component reflects trap-assisted recombination via oxygen-induced surface states. Minor oscillations after the rise edge (Figure 3e,f insets) arise from measurement circuit effects [38], with graphene showing reduced oscillations compared with Au, suggesting improved contact characteristics. The similar decay constants (~865–889 ns overall) for both electrode types indicate that carrier relaxation is governed by diamond’s intrinsic properties rather than by the electrode material, confirming that graphene enhances carrier generation and collection without introducing additional recombination centers.

**Figure 4:**
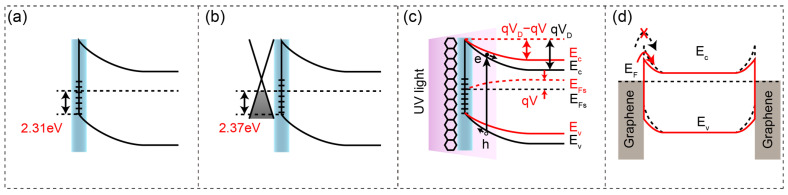
Photo-modulation mechanism. (**a**,**b**) Energy band diagrams derived from XPS showing Fermi levels at 2.31 eV and 2.37 eV above VBM, respectively. The blue vertical bars indicate the oxygen-terminated surface states at the diamond surface. (**c**) Photo-modulation mechanism: photogenerated carriers create an opposing electric field. E_Fs_: Fermi level of diamond; qV_D_: potential barrier height on the diamond side; V: photon-induced voltage; E_C_: conduction-band bottom; E_V_: valence-band top. (**d**) Back-to-back Schottky configuration under dark (dashed) and illuminated (solid) conditions. The dashed configuration also represents Au electrode devices under both conditions due to their UV opacity.

To reveal the band engineering mechanism underlying device performance improvement, the Fermi level alignment was investigated through XPS measurements. The Fermi level positions relative to the valence band maximum (VBM) were determined from XPS C 1s binding energies using the relation E_F_ = BE_C1s_ − 284.01 eV, where 284.01 eV is the fixed energy separation between the C 1s core level and VBM in diamond [39,40]. For bare oxygen-terminated diamond, E_F_ = 2.31 eV above VBM (BE_C1s_ = 286.32 eV, Figure 2f). Following graphene integration, E_F_ = 2.37 eV above VBM (BE_C1s_ = 286.38 eV, Figure 2g). Based on our previous systematic investigation of oxygen-terminated diamond energy band structures [41], the energy band diagrams shown in Figure 4 were constructed by combining experimental XPS measurements of the surface Fermi level with theoretical calculations of the bulk Fermi level. Energy band diagrams reveal that graphene integration (Figure 4b) maintains the upward band bending and depletion region established by oxygen termination (Figure 4a), with only a 0.06 eV Fermi level shift, confirming preserved surface state pinning and built-in field strength.

Under UV illumination, the Schottky barrier is dynamically reduced through the photovoltaic effect (Figure 4c). When UV photons penetrate the graphene electrode, they generate electron–hole pairs near the interface. The built-in electric field separates these carriers: electrons drift toward the diamond bulk, while holes accumulate at the surface. This charge redistribution creates a photoinduced electric field opposing the original built-in field, effectively lowering the barrier height. In the back-to-back electrode configuration (Figure 4d), photo-modulation mechanisms yield a device with optimal performance characteristics: high Schottky barriers in the dark (dashed lines) ensure ultralow dark current, while photo-reduced barriers under UV illumination (solid lines) enhance carrier collection efficiency and responsivity.

**Figure 5:**
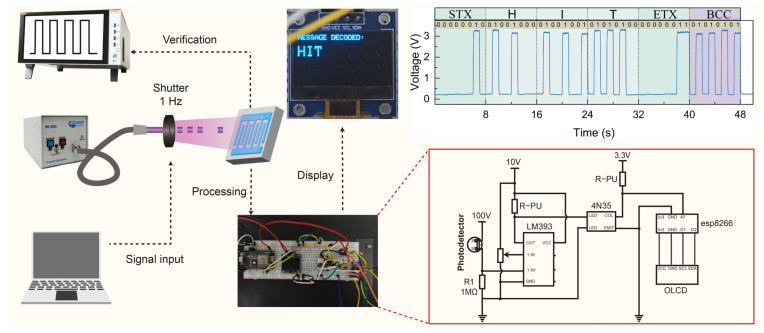
UV optical communication system demonstration using the graphene-electrode diamond photodetector.

To demonstrate the practical utility of the graphene-electrode diamond UV photodetector in real-world applications, a complete UV optical communication system was developed, as illustrated in Figure 5. This system leverages the enhanced photoresponse and faster rise time of optimized device to enable reliable data transmission using ultraviolet light as the carrier.

The communication system consists of four main functional blocks: signal input, optical transmission, signal processing, and display. A computer-generated digital signal modulates a deuterium UV lamp through a mechanical shutter operating at 1 Hz frequency, creating binary on–off patterns that represent encoded information. The photodetector converts these optical signals into electrical current variations, which are then transformed into voltage signals through a voltage divider network. These voltage signals are then fed into an LM393 comparator, which generates clean digital pulses by comparing the input voltage with a predetermined threshold. This approach effectively minimizes the impact of noise and baseline fluctuations, ensuring reliable signal recovery even under variable ambient conditions. A key design feature is the 4N35 optocoupler, which provides essential electrical isolation between the high-voltage detection circuitry and the low-voltage ESP8266 microcontroller, enhancing system safety and reliability. The microcontroller decodes the signals and displays the recovered message on an OLED screen. The system operated at a data rate of 1 bit per second, limited by the maximum frequency of the mechanical shutter rather than by the photodetector itself. Given the detector’s measured rise time of 30 ns, the device theoretically supports data rates up to several MHz. The lower portion of Figure 5 displays the voltage–time trace recorded during the communication process, showing distinct high and low states corresponding to the transmitted binary sequence. The message is structured using standard communication protocols with start-of-text (STX) and end-of-text (ETX) markers, along with block check character (BCC) error-checking codes to ensure transmission integrity. This proof-of-concept system highlights the practical value of the photodetector for secure, solar-blind communication applications that remain unaffected by ambient visible light interference. Real-time operation of the UV communication system is demonstrated in Supplementary Materials Video S1.

## 4. Conclusions

Enhanced deep-UV photodetection was demonstrated by integrating monolayer graphene electrodes with oxygen-terminated diamond. Compared with conventional Au electrodes, graphene-based devices achieved a 7.8-fold photocurrent enhancement, a 4.9-fold responsivity improvement (0.158 A/W at 220 nm), and a 5.2-fold detectivity increase (8.35 × 10^13^ cm·Hz^1/2^/W) while maintaining ultralow dark current (~10^−12^ A). The 27% faster response (30 ns vs. 41 ns) and bi-exponential decay (τ_1_ ≈ 75 ns, τ_2_ ≈ 411 ns) reflect band-to-band recombination and trap-assisted recombination via oxygen-terminated surface states. This enhancement stems from photo-modulation at the graphene–diamond interface: UV photons penetrating the atomically thin graphene dynamically reduce Schottky barriers through photoinduced fields, while maintaining high barriers under dark conditions. The devices showed excellent ambient stability and successfully demonstrated UV optical communication capability. This transparent electrode strategy resolves the responsivity–dark current trade-off in diamond photodetectors, offering a versatile approach for wide-bandgap semiconductor optoelectronics with applications in secure communications and environmental sensing.

## Data Availability

The raw data supporting the conclusions of this article will be made available by the authors on request.

## Supplementary Materials

The following supporting information can be downloaded at https://www.mdpi.com/article/10.3390/s25247536/s1: Video S1: Real-time operation of the UV communication system.
